# A Compliant Force Control Scheme for Industrial Robot Interactive Operation

**DOI:** 10.3389/fnbot.2022.865187

**Published:** 2022-03-23

**Authors:** Xianfa Xue, Haohui Huang, Lei Zuo, Ning Wang

**Affiliations:** ^1^Key Laboratory of Autonomous Systems and Networked Control, School of Automation Science and Engineering, South China University of Technology, Guangzhou, China; ^2^Department of Automation, Shanghai Jiao Tong University, Shanghai, China; ^3^School of Electronic and Control Engineering, Chang'an University, Xi'an, China; ^4^Bristol Robotics Laboratory, The University of the West of England, Bristol, United Kingdom

**Keywords:** single point demonstration, DMP generalization, adaptive compliant control framework, position deviation, grinding experiments

## Abstract

To meet the enormous demand for smart manufacturing, industrial robots are playing an increasingly important role. For industrial operations such as grinding 3C products, numerous demands are placed on the compliant interaction ability of industrial robots to interact in a compliant manner. In this article, an adaptive compliant control framework for robot interaction is proposed. The reference trajectory is obtained by single-point demonstration and DMP generalization. The adaptive feedforward and impedance force controller is derived in terms of position errors, and they are input into an admittance controller to obtain the updated amount of position deviation. The compliant interaction effect is achieved, which is shown that the grinding head fits on the curved surface of a computer mouse, and the interaction force is within a certain expected range in the grinding experiment based on the performance an Elite robot. A comparative experiment was conducted to demonstrate the effectiveness of the proposed framework in a more intuitive way.

## Introduction

With the rapid development of industry, the traditional model cannot meet the demand of high-speed production, and as a result industrial robots are necessary to change the original production model. In the industrial 4.0 age, industrial robots in intelligent manufacture have attracted more and more attention (Qi et al., 2020; Cheng et al., 2021; Lu et al., 2021). In recent years, robot technology has broad application prospects in many fields, most of the working procedures in factories have long been replaced by industrial robots (Luo et al., 2020; Su et al., 2020). In the processes of intelligent manufacturing, such as grinding steam turbine engine blades, polishing helicopter propellers, automobile component assembly, and so on, it is impossible to realize these precise and meticulous operations through only the traditional position control and force control. Therefore, the research on the compliant force control of industrial robots has great practical value and engineering significance (Chao, 2017; Zhang K. et al., 2021).

Compliance can be divided into active compliance and passive compliance (Kim et al., 2010). Passive compliance relies on some auxiliary compliance mechanisms; active compliance means that the robot uses the information feedback at the end and adopts a certain control strategy to produce an active control force (Wang et al., 1998; Zhu et al., 2021). Due to the engineering value of compliance control, it has attracted extensive attention from scholars. Some researchers use machine learning methods based on Gaussian mixture models (GMM) and artificial neural network models to model motion and force using temporal information (Yun et al., 2012; Müller et al., 2020). The development of vision sensors and force sensors also provides great convenience for compliance control (Lee et al., 2012; Niu et al., 2021). Following the obedience behavior mechanism of the human arm also provides a new control strategy and idea for the robot's compliant force control (Zeng et al., 2022). Due to the high-performance requirements of robot position control and force control in grinding, engraving, and other operations, the current research level on compliance control is insufficient (Park et al., 2008; Zhou et al., 2021).

In this study, a complete framework of a compliant control scheme for industrial robot interactive operation is developed. Firstly, the reference trajectory is obtained by single point teaching and DMP generalization. Then the motion error information is input into the adaptive feedforward and impedance force controller. Next, they are input into the admittance controller to obtain the updated amount of position deviation. Finally, the motion of the manipulator is controlled by the robot joint controller. The framework can adaptively adjust the reference trajectory and interaction force, which greatly enhances the interaction accuracy. The contributions can be summarized as follows. (1) A complete framework of an adaptive compliant control scheme for industrial robot interactive operation is developed to take into account the feedforward force and impedance force, which guarantees the robot end effector to better fit on the interacting surface and the interaction force to be within a certain expected range. (2) The framework is applied to the grinding task with rigorous requirements for the control accuracy of position and force, which achieves a good grinding effect.

The rest of the article structure is organized as follows. In methodology, the methods of reference trajectory generation and adaptive compliant controller used in this article are introduced. In experiment and result, the experimental study is presented and then the effectiveness of the framework proposed in this article is verified via computer mouse surface grinding experiments. In discussion, the experimental results are analyzed and explained. Finally, the conclusions section summarizes the whole article.

## Methodology

### Overview of the Framework

**Figure 1:**
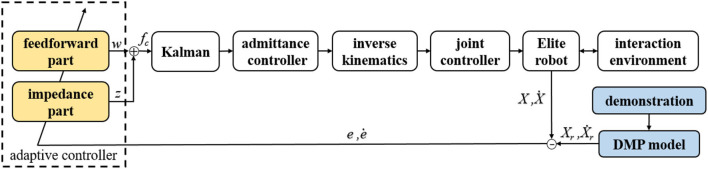
Overview of the proposed framework.

The block diagram of the proposed framework is shown in Figure 1. In the proposed compliant interaction framework, the human tutor presents a demonstration at first. The trajectory learned from the DMP model is regarded as the reference trajectory *Xr*. Then the motion error information is input into the adaptive feedforward and impedance force controller. After removing part of the disturbance, they are input into the admittance controller to obtain the updated amount of position deviation. Finally, the motion of the manipulator is controlled by the robot joint controller.

### Preliminaries and Control Methods

#### Dynamic Movement Primitives

In this article, the DMP model is used to generate the motion trajectory (Liu N. et al., 2020). The principle and main calculation process of the DMP model are as follows (Ude et al., 2014).

A single degree of freedom motion DMP model can be expressed by a spring-damping nonlinear dynamical second-order system as follows.


(1) $$\begin{array}{r} \xi {\dot{\Upsilon }}_{2}=a\left(g-\Upsilon_{1}\right)-b\Upsilon_{2}+\Gamma \left(s,\omega \right) \end{array}$$

(2) $$\begin{array}{r} \xi {\dot{\Upsilon }}_{1}=\Upsilon_{2} \end{array}$$

(3) $$\begin{array}{r} \xi ṡ=-k_{1}s \end{array}$$

where, in order to simplify the model, the time variable is ignored. Υ_1_(*t*) is represented by Υ_1_; the system spring and damping coefficient are represented by *a* and *b* respectively. We set *a* = *b*^2^/4. *g* is the desired value of the motion trajectory. ξ indicates the time scaling coefficient. Υ_1_ and Υ_2_ represent the position and velocity of motion trajectory, respectively. ω represents the weight of the Gaussian model. In Equation (3), *s* represents the phase variable of the system, and *k*_1_ is a positive constant. The nonlinear function Γ(*s*, ω) is defined as follows.


(4) $$\begin{array}{r} \Gamma \left(s,\omega \right)=\frac{\sum_{i=1}^{N}\psi_{i}\omega_{i}}{\sum_{i=1}^{N}\psi_{i}}\left(g-\Upsilon_{0}\right)s \end{array}$$

(5) $$\begin{array}{r} \psi_{i}=\exp\left(-d_{i}{\left(s-c_{i}\right)}^{2}\right) \end{array}$$

where, ω_*i*_ *d*_*i*_ and *c*_*i*_, are the weight, width, and center of the *i*-th kernel function, respectively. Υ_0_ is the initial value of the motion trajectory; *N* is the total number of Gaussian models (Zhang Y. et al., 2021).

We set the initial value of *s* as 1, which decays to 0 in the process of time. The nonlinear function Γ(*s*, ω) is bounded because the value of *s* tends toward 0. Thus, the model is a stable second-order spring-damping system.

In this algorithm, the local weighted regression algorithm (LWR) is used to determine model parameters ω. The demonstration trajectory Υ(*t*) is obtained, where *t* = [1, 2..., *T*], *g* = Υ(*T*) (Lin et al., 2020).

#### Gravity Compensation and Force Sensor Calibration

In applications of industrial robots such as grinding, the contact force between the end tool of the robot and the external environment needs to be accurately perceived, and the control system modifies the motion of the robot accordingly in order to ensure compliance of the operation. Therefore, gravity compensation is very necessary (Yang et al., 2020; Yu et al., 2021).

On the basis of ensuring the sensor installation angle through mechanical positioning, using the sensor data under the general attitude of no <3 robots, the parameters such as sensor zero point, robot installation inclination, load gravity, and load gravity center coordinates are obtained by using the least square method. The specific calculation process is referred to Zhang et al. (2017). Finally, the real force in X, Y, and Z directions after gravity compensation is


(6) $$\begin{array}{r} F_{sx}=F_{x}-F_{x0}-G_{x} \end{array}$$

(7) $$\begin{array}{r} F_{sy}=F_{y}-F_{y0}-G_{y} \end{array}$$

(8) $$\begin{array}{r} F_{sz}=F_{z}-F_{z0}-G_{z} \end{array}$$

where, *F*_*x*_, *F*_*y*_, and *F*_*z*_ are the three force components directly measured by the force sensor; *F*_*x*0_, *F*_*y*0_, and *F*_*z*0_ are the sensor zero values obtained after identification; *G*_*x*_, *G*_*y*_, and *G*_*z*_ represent the components of the load gravity in the X, Y, and Z directions of the force sensor coordinate, respectively.

#### Kalman Filter

The force measured by the F/T sensor usually contains a lot of noise, which can seriously affect the control accuracy. Considering the good performance and simple implementation of the Kalman filter, in this article, we choose the Kalman filter for noise reduction to obtain a more accurate input force (Tsai et al., 2010; Liu J. et al., 2020). At first, we suppose that the noise is Gaussian white noise. The system and measurement model can be defined as Equations (9, 10).


(9) $$\begin{array}{r} \dot{𝔛}\left(t\right)=A_{0}𝔛\left(t\right)+U \end{array}$$

(10) $$\begin{array}{r} 𝔜\left(t\right)=B_{0}𝔛\left(t\right)+C_{0}g+V \end{array}$$

where, U and V are system and measured noise, respectively (Zhu et al., 2018).

We discrete the continuous system and yield the different equations as follows.


(11) $$\begin{array}{r} {𝔛}_{k}=A{𝔛}_{k-1}+U_{k-1} \end{array}$$

(12) $$\begin{array}{r} {𝔜}_{k}=B{𝔛}_{k}+Cg+V_{k} \end{array}$$

(13) $$\begin{array}{r} A=\left[\begin{array}{cc} I & I\\ 0 & I \end{array}\right],B=B_{0},C=C_{0} \end{array}$$

The state ${\hat{𝔛}}_{k|k-1}^{-}$ and the covariance $P_{k|k-1}^{-}$ at time step *k* decided by the result at time step *k*-1 can be calculated as follows, where *S* and *M* indicate the covariance of the system and measured noise, respectively.


(14) $$\begin{array}{r} {\hat{𝔛}}_{k|k-1}^{-}=A{\hat{𝔛}}_{k-1} \end{array}$$

(15) $$\begin{array}{r} P_{k|k-1}^{-}=AP_{k-1}A^{T}+S \end{array}$$

The gain *G*_*k*_ and the optimal estimation at time step *k* is obtained by:


(16) $$\begin{array}{r} G_{k}=P_{k|k-1}^{-}B^{T}{\left(BP_{k|k-1}^{-}B+M\right)}^{-1} \end{array}$$

(17) $$\begin{array}{r} {\hat{𝔛}}_{k}={\hat{𝔛}}_{k|k-1}^{-}+G_{k}\left({𝔜}_{k}-B{\hat{𝔛}}_{k|k-1}^{-}\right) \end{array}$$

The covariance $P_{k}$ is updated at last:


(18) $$\begin{array}{r} P_{k}=\left(I-G_{k}B\right)P_{k|k-1}^{-} \end{array}$$

#### Robot Dynamic Description

The dynamics of the robot in the joint space is as follows (Murray et al., 2018; Ahmad et al., 2021).


(19) $$\begin{array}{r} M_{q}\left(q\right)\ddot{q}+C_{q}\left(q,\dot{q}\right)\dot{q}+G_{q}\left(q\right)=\tau_{c}+\tau_{ext} \end{array}$$

where *q*, $\dot{q}$, and $\ddot{q}$ are the coordinate, speed, and acceleration in the joint space, respectively. $M_{q}\left(q\right)\in R^{n\times n}$ is the inertia matrix of the manipulator, $C_{q}\left(q,\dot{q}\right)\in R^{n\times n}$ represents the centrifugal and Coriolis force vector, $G_{q}\left(q\right)\in R^{n\times 1}$ represents the gravity torques, $\tau_{c}\in R^{n}$ is the vector of the control input, and $\tau_{ext}\in R^{n}$ is the vector of the measured interaction force, which is the force exerted by the human on the robotic arm.

Since the manipulator is controlled in Cartesian space, the above formula is transformed from joint space to Cartesian space as follows:


(20) $$\begin{array}{r} M_{x}\left(q\right)ẍ+C_{x}\left(q,\dot{q}\right)ẋ+G_{x}\left(q\right)=f_{c}+f_{ext} \end{array}$$

where *x*, ẋ, and ẍ are the coordinate, speed, and acceleration in Cartesian space, respectively. The remaining variables are transformed from the joint space to Cartesian space as follows, and robot dynamics have the following two properties.


(21) $$\begin{array}{r} M_{x}=J^{-T}M_{q}J^{-1} \end{array}$$

(22) $$\begin{array}{r} C_{x}=J^{-T}\left(C_{q}-M_{q}J^{-1}\dot{J}\right)J^{-1} \end{array}$$

(23) $$\begin{array}{r} G_{x}=J^{-T}G_{q} \end{array}$$

(24) $$\begin{array}{r} f_{c}=J^{-T}\;\tau_{c} \end{array}$$

(25) $$\begin{array}{r} f_{ext}=J^{-T}\;\tau_{ext} \end{array}$$

Property 1: *M*_*x*_(*q*) is a symmetric and positive definite matrix.

Property 2: $2C_{x}\left(q,\dot{q}\right)-{Ṁ}_{x}\left(q\right)$ is a skew-symmetric matrix.

#### Admittance Controller

The prescribed admittance model is defined as follows:


(26) $$\begin{array}{rcl} M_{E}\left(Ẍ-{Ẍ}_{r}\right) & + & C_{E}\left(Ẋ-{Ẋ}_{r}\right)+K_{E}X=f_{in} \end{array}$$

(27) $$\begin{array}{r} f_{in}=f_{c}-f_{R} \end{array}$$

Where, *f*_*in*_ is the input of admittance controller; *f*_*c*_ is robot drive force; *f*_*R*_ is the desired reference interaction force. *X*, Ẋ, and Ẍ represent the current position, velocity, and acceleration, respectively. Ẋ_*r*_ and Ẍ_*r*_ represent the reference velocity and acceleration, respectively. *M*_*E*_, *C*_*E*_, and *K*_*E*_ represent the unknown mass, damping, and stiffness matrices in the model, respectively. However, since the mass matrix *M*_*E*_ is usually high nonlinear. In this study, the mass-damping-stiffness model is simplified as the damping-stiffness model, which is used to interact with a balloon as a kind of flexible object (Huang et al., 2020; Shen et al., 2020). The simplified model is as follows:


(28) $$\begin{array}{r} C_{E}\left(Ẋ-{Ẋ}_{r}\right)+K_{E}X=f_{c}-f_{R} \end{array}$$

#### Adaptive Control Model

Inspired by the human arm motor learning mechanism, we can think that the control input *f*_*c*_ consists of the sum of a feedback term and a feedforward term.


(29) $$\begin{array}{r} f_{c}=z+w \end{array}$$

where *z* and *w* are the feedback force (impedance term) and the feedforward force, respectively. The impedance term is described as follows.


(30) $$\begin{array}{r} z=Dė+Ke \end{array}$$

(31) $$\begin{array}{r} e=X_{r}-X \end{array}$$

(32) $$\begin{array}{r} ė=\dot{X_{r}}-Ẋ \end{array}$$

where *e* and ė are the auxiliary displacement error vector and the auxiliary velocity error vector, respectively. *D* and *K* represent the endpoint damping matrix and stiffness matrix, respectively.

The stiffness matrix elements will be adapted according to the task situation. Then we can calculate the damping matrix by the stiffness matrix as $D_{i}^{t}=\varsigma \sqrt{K_{i}^{t}}$ at each time step. At the beginning, we predefine the positive coefficient ς and the constant *i* = [1, 2, 3].

We choose the following cost function for the concurrently minimizing of the motion trajectory error and the effort (Ganesh et al., 2010):


(33) $$\begin{array}{r} J_{cost\;}=\frac{\alpha }{2}z^{T}z+\overset{N}{\underset{i=1}{\sum }}\gamma_{i}w_{i} \end{array}$$

where α and γ are the *N*-dimensional parameter vectors. α > 0 and γ > 0 are used to calculate the corresponding endpoint impedance force and feedforward force, respectively. The former term indicates the cost of motion error, and the latter term, i.e., the weighted sum of the feedforward items, indicates the cost of effort (Ye et al., 2021).

We suppose the impedance term *z* as a linear function, increasing in both negative and positive directions. This supposition is according to the mechanism of human motor learning, which can be described as follows (Zeng et al., 2020).


(34) $$\begin{array}{r} z_{i}=\varepsilon_{i,+}+\zeta \varepsilon_{i,-},\zeta \in \left(0,1\right) \end{array}$$

where, ε_*i*, +_ and ε_*i*, −_ represent the negative and positive directions, respectively, and they are defined as follows.


(35) $$\begin{array}{r} \varepsilon_{i}=\pi \left(e_{i}+\delta \dot{e_{i}}\right) \end{array}$$

(36) $$\begin{array}{r} \varepsilon_{i,+}=\max\left(\varepsilon_{i},0\right) \end{array}$$

(37) $$\begin{array}{r} \varepsilon_{i,-}={\left(-\varepsilon \right)}_{i,+} \end{array}$$

where, π and δ are positive constants, and ε represents the sliding error vector.

The learning of Equation (33) can be defined as a gradient descent problem, which can be described as follows.


(38) $$\begin{array}{r} \Delta w^{t}=w^{t+1}-w^{t}=-\frac{\partial J_{cost\;}}{\partial w} \end{array}$$

Then yielding the following law:


(39) $$\begin{array}{r} \Delta w^{t}=\alpha z^{t}-\gamma \end{array}$$

According to Equations (34) and (35), we divide the adaptation law into three terms: an antisymmetric term, a symmetric term, and a bias term.


(40) $$\begin{array}{r} \Delta w^{t}=\frac{\alpha }{2}\left(1-\zeta \right)\varepsilon^{t}+\frac{\alpha }{2}\left(1+\zeta \right)|\varepsilon^{t}|-\gamma \end{array}$$

## Experiment and Result

**Figure 2:**
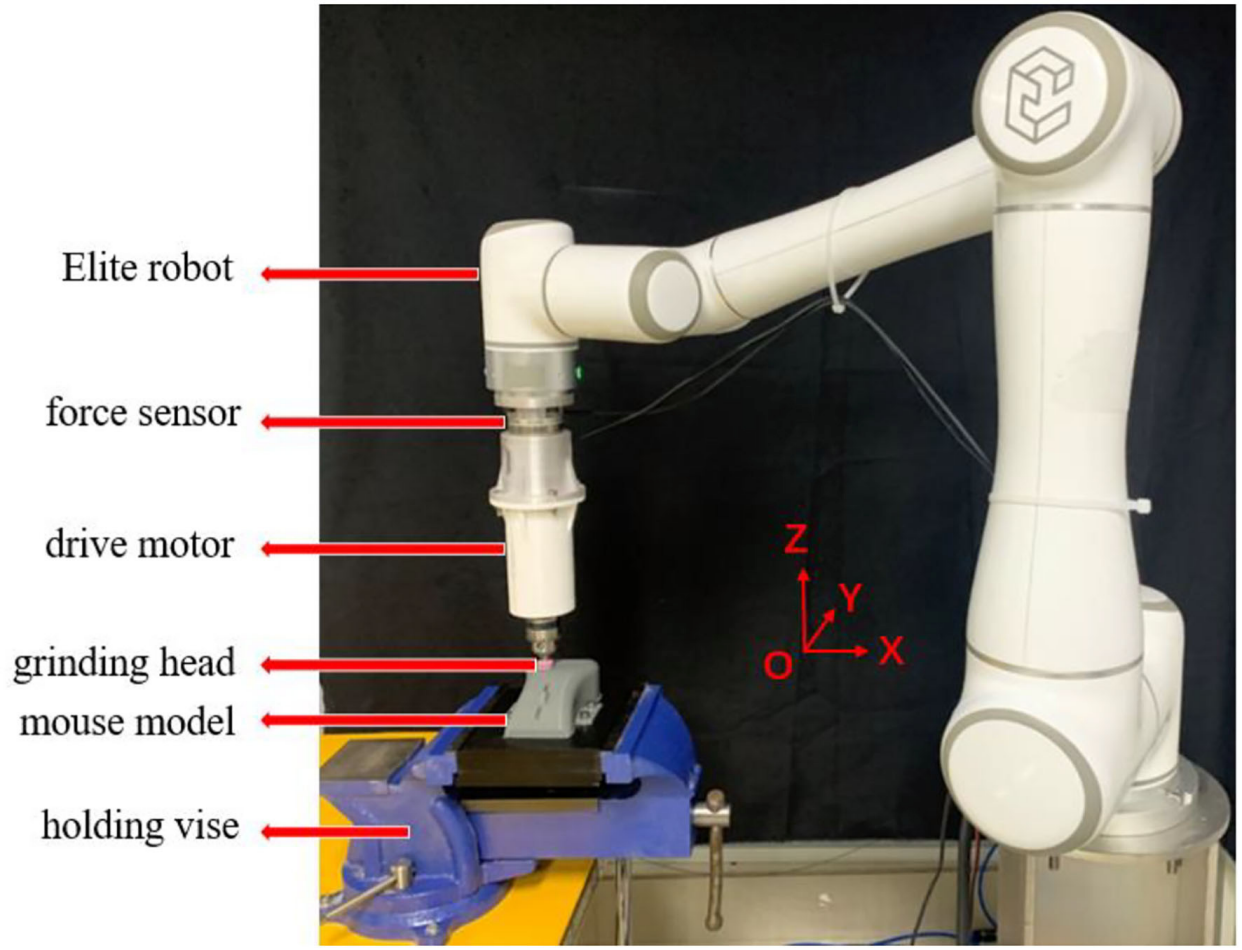
Grinding experiment platform based on Elite robot.

In this section, the performance of the proposed compliant force control scheme was validated by conducting the experiment on a 6 degree of freedom Elite-EC66 robot as shown in Figure 2. The ATI Mini45 Force/Torque sensor was mounted on the end of the manipulator through the connecting flange to sense the interacting force between the end effecter and the environment in real-time. The computer mouse model to be ground was fixed on the experiment bench by a holding vice. The mouse model used in the experiment was made by 3D printing, and its material was PETG polymer. The side view of the mouse model is shown in **Figure 4A**. A cylindrical sand grinding head with a diameter of 1 cm was driven to rotate by a drive motor. The rated power of the drive motor was 80 w and the rotating speed was 5,000 r/min. The force sensor and the upper computer communicated by the UDP protocol whose sampling rate and control rate was set as 100 and 50 Hz, respectively.

### Gravity Compensation Effect Test

**Figure 3:**
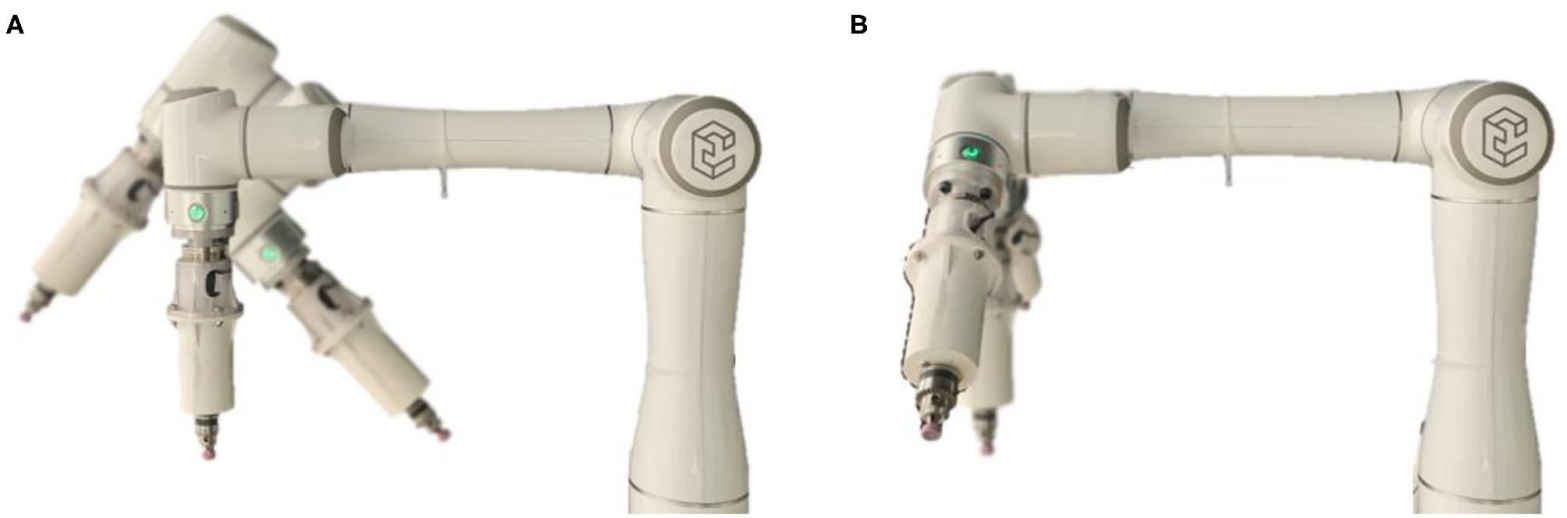
Force compensation test with different end positions and angles. **(A)** Posture transformation in the Y-O-Z plane. **(B)** Posture transformation in the Z-O-X plane.

In order to more accurately reflect the interactive force information between the grinding head and the mouse, it is necessary to compensate for the zero point of the sensor and the gravity of the end external tool. According to the method in Section Gravity Compensation and Force Sensor Calibration, we tested the force compensation effect. The weight of the end tool identified by the algorithm was 790.33 g, and the two installation inclination angles of the Elite robot were −4.34 and −1.35°, respectively. The zero point of the force sensor was: (*F*_*x*0_, *F*_*y*0_, *F*_*z*0_) = (−0.13, 0.76, −7.91) N. As shown in Figure 3, we tested the effect of force compensation for different end postures. In the case of no external environment interaction, the following five representative gestures in the Y-O-Z plane and the Z-O-X plane were selected for verification. The result data were shown in Table 1.

**Table 1:** Force compensation testing results in the case of no external environment interaction.

**End postures**	**Forces in base coordinate**
0°	(0.06, −0.06, −0.22) N
45° (Y-O-Z)	(1.21, 0.87, −0.49) N
−45° (Y-O-Z)	(0.95, −0.69, 1.13) N
45° (Z-O-X)	(−0.98, −1.20, 0.89) N
−45° (Z-O-X)	(1.02, 0.91, −1.07) N

### Grinding Trajectory Generation

**Figure 4:**
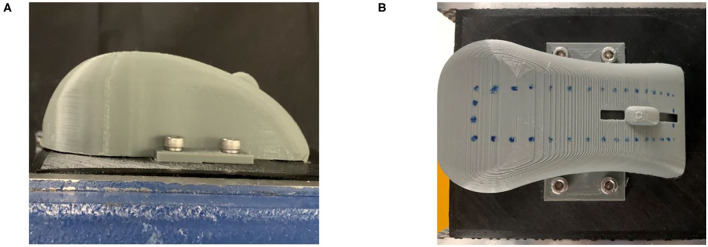
**(A)** Side view of the mouse model to be ground. **(B)** Distribution of demonstration points on mouse curved surface.

The grinding trajectory was generated by DMP generalization after human teaching of the robot. Due to the function limitation of the Elite manipulator, it was difficult to demonstrate through continuous dragging. In the teaching stage, the single-point teaching method was adopted for the elite robot. As shown in Figure 4B, we marked the teaching points on the mouse surface with a marker pen. The human tutor dragged the robot arm to demonstrate 16 points on the long side and 4 points on the short side. In the meantime, the teaching points information was recorded through the program.

**Figure 5:**
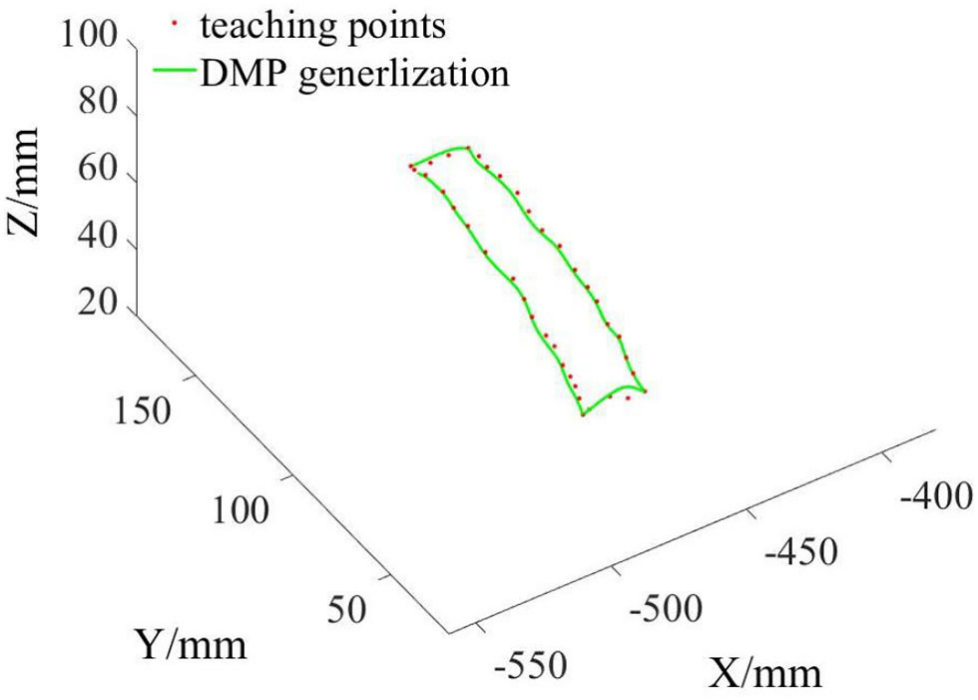
Desired grinding trajectory generating via demonstration and DMP.

The teaching points were divided into four segments and put into the DMP model for training. After that, the four trajectories interpolated and generalized by DMP were spliced into one trajectory as the grinding reference trajectory *X*_*r*_. The three-dimensional space graph of the desired grinding trajectory is shown in Figure 5.

### Compliant Grinding Experiments

**Figure 6:**
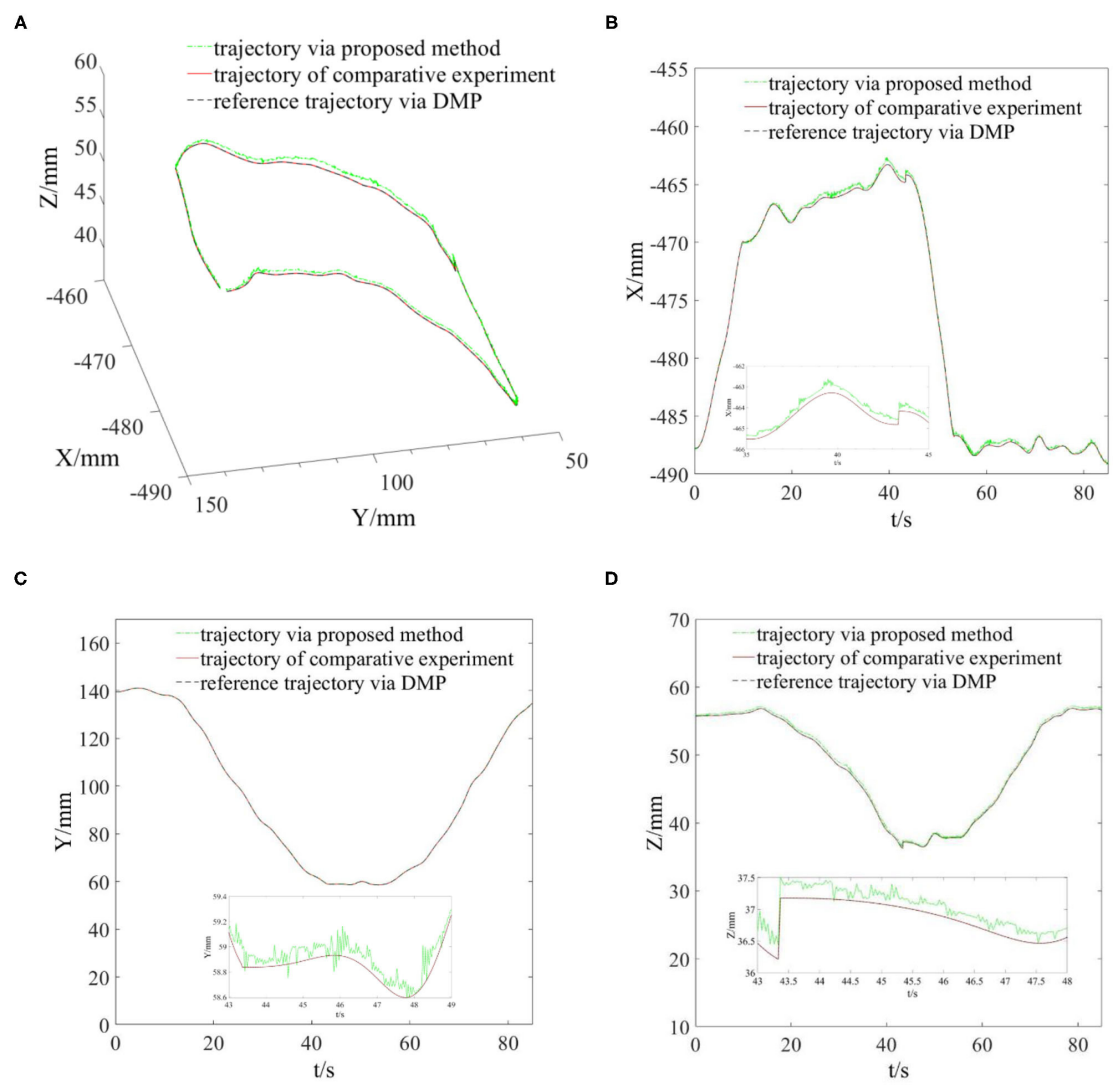
Reference and actual grinding trajectories of two experiments. **(A)** Spatial view of grinding trajectories. **(B)** Grinding trajectories in the X direction. **(C)** Grinding trajectories in the Y direction. **(D)** Grinding trajectories in the Z direction.

**Figure 7:**
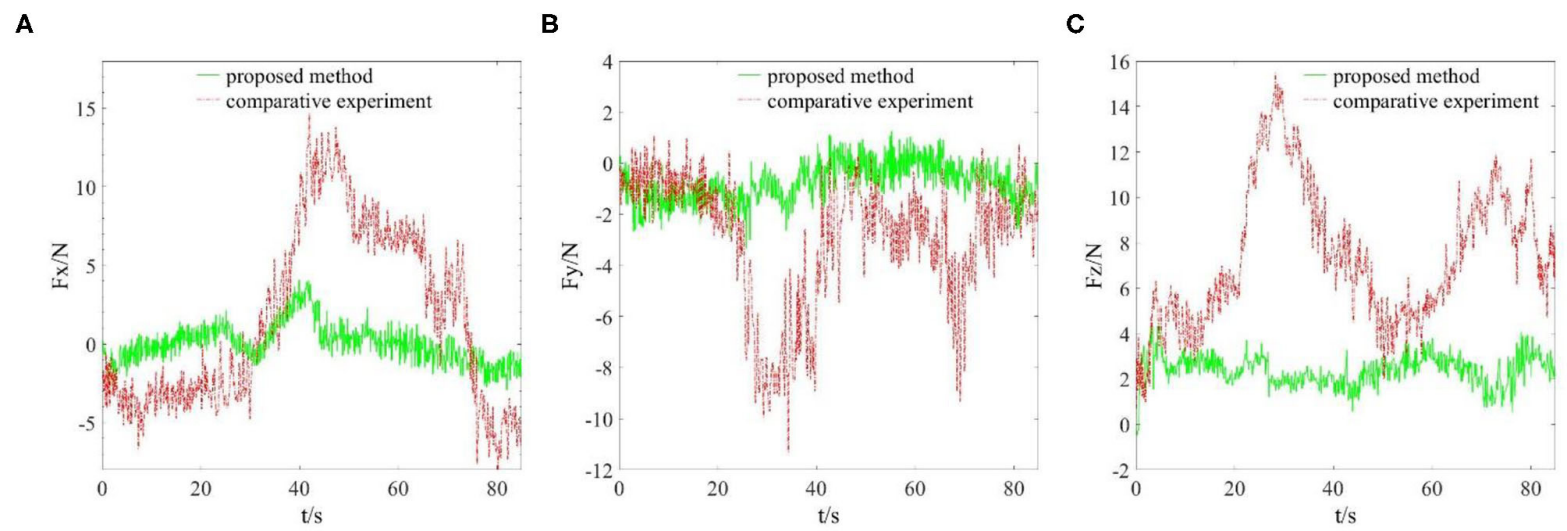
Grinding interaction forces in X, Y, and Z directions. **(A)** Interaction force in X direction. **(B)** Interaction force in Y direction. **(C)** Interaction force in Z direction.

In this part, we conducted grinding experiments according to the reference trajectory generated in the previous part, and monitored the actual grinding trajectory and grinding interaction force. The main parameters in the experimental program were set as: π = 1.7; δ = 0.02; α = [10, 10, 10]^T^; γ = [5, 5, 5]^T^; *C*_*E*_ = diag [4, 4, 4]; *K*_*E*_ = diag [20, 20, 20]. The grinding time to complete the reference trajectory was 85 s. Due to the proposed algorithm being able to adjust the position, the end grinding head trajectory did not completely follow the reference trajectory in the grinding process. In order to more intuitively see the effect of the compliant grinding framework proposed in this article, we also conducted another comparative experiment. In the comparative experiment, we removed the compliant force control scheme part and only carried out the grinding experiment under the control of the joint controller of the Elite robot. To meet the conditions of the two experiments as much similar as possible, we used the same reference trajectory for grinding. We replaced the mouse model with a fresh copy, and it is worth mentioning that the mouse models used in the two experiments are based on the same model and from the same 3D printer. That is, their sizes and materials are identical. Similar to the first experimental process, we also recorded the actual trajectories in the X, Y, and Z directions of the end grinding head. Then, we draw the reference grinding trajectory, the compliant grinding trajectory, and the comparative experimental grinding trajectory in the same coordinate system, as shown in Figure 6. During two grinding experiments, the grinding interaction forces in X, Y, and Z directions were recorded at the same time, as shown in Figure 7.

**Figure 8:**
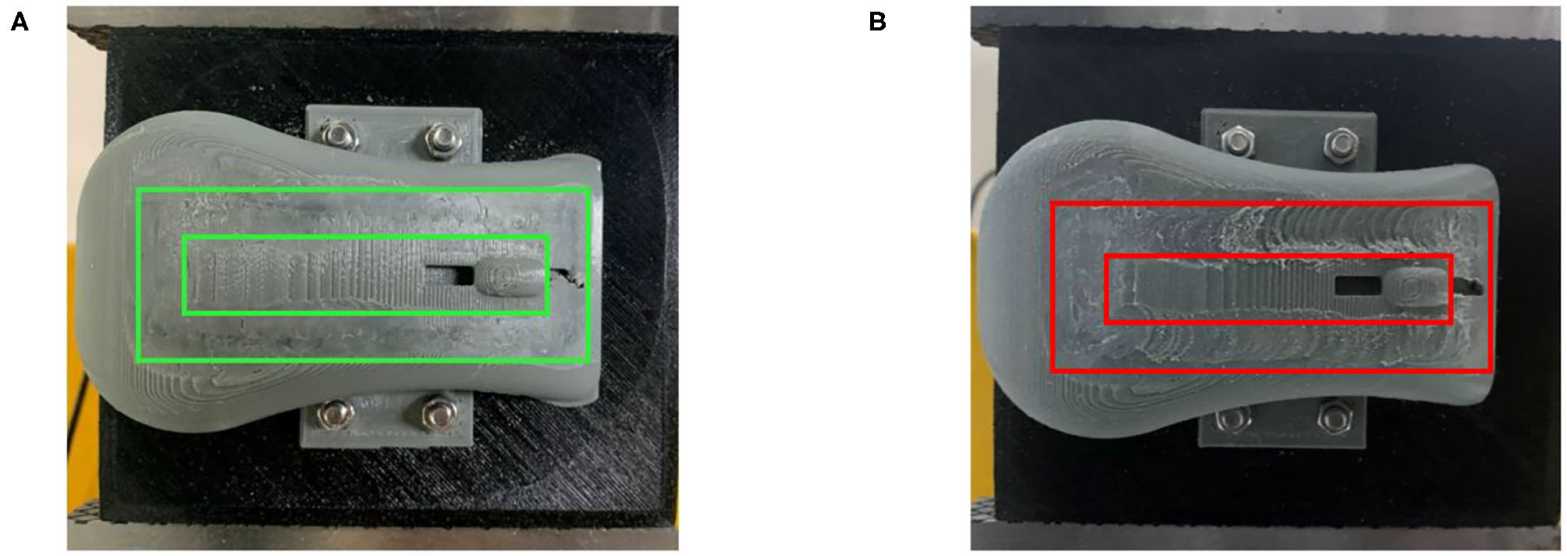
**(A)** Grinding effect based on the compliant grinding framework. **(B)** Grinding effect of the comparative experiment.

After two grinding experiments, the actual grinding effect photos of the mouse models are shown in Figure 8. The annular area between two black rectangles was the grinding effect based on the compliant grinding framework, and the annular area between two red rectangles indicated the grinding effect of the comparative experiment.

## Discussions

From Figure 5, we can see that the grinding trajectory generated by DMP generalization does not completely fit all teaching points. Therefore, we cannot directly use the reference trajectory obtained by the demonstration for position control, especially for grinding tasks, which have high requirements on position and force accuracy. So it is necessary to adjust and update the desired position based on the reference trajectory in combination with the interaction force information. From Figure 6, it is not difficult to see that the compliant grinding framework proposed in this article can calculate the updated amount of position deviation depending on the size of the interaction force, so as to ensure that the grinding head can better fit the surface of the mouse model without excessive extrusion. Figure 7 shows the change in interaction force during compliant grinding. The contact force in the Z-direction fluctuates up and down around 3 N, and the maximum value does not exceed 5 N. However, if the compliant force control part is removed, the degree of force instability and fluctuation increases. Moreover, two peaks of the force in the Z-direction exceed 10 N. It can also be seen from Figure 8B that excessive contact force produces a large amount of grinding heat, and the high temperature makes the mouse surface begin to melt. It is obvious that excessive grinding force will seriously affect the grinding effect of the mouse model. However, there are many disturbances in the grinding process. Kalman filter alone cannot solve the problem of disturbance compensation. In the later research work, we will further study how to better compensate for the grinding disturbances and improve the grinding accuracy.

## Conclusions and Future Work

In this article, a robotic-compliant grinding framework was proposed. With learning from the demonstration approach, the robot can obtain reference input more quickly. The interpolation and generalization of DMP overcomes the difficulty of continuous demonstration. In addition, the feedforward force and impedance force were adaptively adjusted to realize the compliant interaction with the ground workpiece. Then the force information was input into the admittance controller to obtain the updated amount of position deviation, so as to adjust the reference trajectory in real-time. At last, the effectiveness of the proposed framework was verified by the grinding experiment of the mouse model surface. The experiments showed that the frame can make the grinding head close to the computer mouse's curved surface and control the interaction force within a certain expected range. Our research will focus on overcoming various grinding disturbances and we hope to improve the compliant grinding performance of the platform.

## Data Availability Statement

The raw data supporting the conclusions of this article will be made available by the authors, without undue reservation.

## Author Contributions

XX, HH, and NW conceived of the presented idea. XX implemented the framework, conducted the experiment, contributed to manuscript writing, and including the original draft. HH and NW contributed to review and provided critical feedback. LZ helped to revise the article and polish the writing of the manuscript. All authors had read the manuscript and agreed with its content.

## Funding

This work was partially supported by the National Natural Science Foundation of China under Grant No. 61803039.

## Conflict of Interest

The authors declare that the research was conducted in the absence of any commercial or financial relationships that could be construed as a potential conflict of interest.

## Publisher's Note

All claims expressed in this article are solely those of the authors and do not necessarily represent those of their affiliated organizations, or those of the publisher, the editors and the reviewers. Any product that may be evaluated in this article, or claim that may be made by its manufacturer, is not guaranteed or endorsed by the publisher.
